# Innovation in traditional sport: applying the delphi method to strategic designs in basque hand-ball

**DOI:** 10.3389/fspor.2026.1704042

**Published:** 2026-01-30

**Authors:** Eneko Sanchez Mencia, Xabier Gonzalez-Santamaria, Maite Aurrekoetxea-Casaus

**Affiliations:** 1 Department of Sports Sciences and Physical Activity, Faculty of Education and Sport, University of Deusto (Bilbao-Spain), Bilbao, Spain; 2 Department of Social Work and Sociology, Faculty of Social Sciences and Humanities, University of Deusto (Bilbao-Spain), Bilbao, Spain

**Keywords:** basque hand-ball, delphi method, heritage sports, scenario planning, traditional sport

## Abstract

**Introduction:**

This study explores the potential of prospective qualitative methods, specifically the combination of the Delphi technique and scenario planning, to guide innovation processes in traditional sports, using the professional modality of Basque hand-ball as a case study.

**Methods:**

Employing a three-round design, the research gathered evaluations from 23 experts embedded within the sport's ecosystem (including players, referees, coaches, organizers, and media representatives) regarding a series of structured proposals linked to key elements of the game (e.g., scoring systems, service, equipment, rest times).

**Results:**

Findings reveal that the most viable innovations are not necessarily the most disruptive, but rather those that balance spectacle, competitive fairness, and symbolic fidelity to the internal logic of the sport. Proposals such as the “short games” system, time limits between serves, and neutral selection of equipment received strong support and have already been incorporated into the current configuration of the professional league.

**Discussion:**

Furthermore, perceived viability varied according to stakeholder profiles, underscoring the relevance of participatory and deliberative approaches in designing culturally legitimate reforms.

## Introduction

In recent decades, traditional sports have been placed under increasing strain due to a dual demand: the need to adapt to new audiences and formats, and the desire to preserve the cultural heritage that defines them. While most studies on sport innovation have focused on highly professionalized disciplines such as football, basketball, or tennis—where logics of efficiency, profitability, and spectacle predominate (1–3)—this approach proves insufficient for understanding the specific challenges faced by heritage sports. In such contexts, introducing organisational or regulatory changes involves not only technical decisions, but also symbolic negotiations that affect collective identity (4).

Basque hand-ball stands as a paradigmatic example of a sport rooted in community life. Beyond its competitive dimension, it has historically functioned as a cultural founding act in the Basque Country, closely tied to territoriality, belonging, and collective memory (5). Its modern version, which emerged in the nineteenth century, is played in enclosed frontons, where two players or pairs strike a leather ball with their hand, alternating turns until one fails to return it properly (6). Today, there are over 17,000 registered players in Spain—mainly in the Basque Country, Navarre and La Rioja—and the sport maintains considerable visibility: the final of the 2024 professional championship drew a television audience of 193,000 viewers (7, 8).

However, intense competition with mainstream sports has placed Basque hand-ball in a vulnerable position. Various institutions have warned of a risk of decline unless renewal strategies are adopted that retain the interest of new generations without undermining the essence of the game. Experiences in other racquet sports have shown that certain regulatory adjustments—such as the rally point system in badminton (9) or the no-ad scoring system in tennis (10)—can serve as levers for revitalization. Nevertheless, these changes also highlight the dangers of hasty commodification that neglects the symbolic grounding of such practices.

Faced with this dilemma, the present study proposes a participatory and prospective methodological approach to identify, in a deliberative manner, possible scenarios for change in Basque hand-ball. Drawing inspiration from proposals such as that of Lamoureaux et al. (2024), it combines the Delphi method with scenario analysis to systematically gather expert knowledge and construct shared future visions that are both culturally sustainable and technically feasible (11–13).

The main contribution of this study is twofold. Methodologically, it advances the use of qualitative tools in the design of context-sensitive sports policies. Substantively, it offers concrete orientations for transforming a heritage sport without eroding the values that sustain it. In doing so, it aims to contribute both to the academic debate on sport and innovation, and to real-world decision-making processes in settings where culture, play and community are deeply intertwined.

## Theoretical framework

### Sport innovation between functional logic and heritage embeddedness

Innovation in sport has been theorized as a domain with characteristics that cannot be fully captured by generic models of organisational or technological innovation. Unlike innovation in conventional industries, innovation in sport unfolds within institutionalized competitive systems characterized by formal rules, embodied performance, and strong symbolic attachments among participants and audiences. As Glebova and Desbordes (14) demonstrate in their typology of technological innovation in sport, sport-related innovations are shaped by environmental, organisational and internal factors that are specific to sporting ecosystems and that directly affect both athletic performance and spectators’ experience.

Building on this sport-specific understanding, sport management research has argued that innovation in sport must be approached as a multi-level process—encompassing technologies, organisational arrangements, competition formats, and governance mechanisms—whose trajectories are conditioned not only by functional performance but also by regulatory frameworks and stakeholder legitimacy (15, 16). In this sense, sport innovation cannot be evaluated solely through efficiency or productivity criteria, as it intervenes directly in norm-governed competitions where fairness, comparability, and legitimacy are constitutive values.

Framing innovation in sport in this way provides the conceptual basis for distinguishing sport-specific criteria of evaluation from generic organisational or technological innovation models, particularly in contexts where cultural meaning and tradition play a central role. This distinction becomes especially relevant in disciplines where sporting practices are closely tied to collective identity and social memory, and where innovation may be perceived as threatening if it disrupts the symbolic coherence of the game.

Empirically, racquet ball sports have undergone profound organisational and regulatory transformations, driven by the need to adapt to increasingly diverse, demanding and fragmented audiences (17). These transformations have included modifications to scoring systems, the introduction of officiating technologies, the reduction of downtime, the reorganization of calendars, and the adoption of more attractive formats for digital audiences (18). Such processes respond not only to the improvement of spectator experience, but also to growing competition from alternative leisure forms, the pressure to ensure sustainable models of sport practice, and the imperative for visibility in an oversaturated media landscape (19).

Nevertheless, this evolution raises significant challenges: how to innovate without diluting the symbolic value of the game; how to preserve identity-defining elements without lapsing into immobility; and how to guarantee equity among the different stakeholders within the sporting ecosystem from athletes and federations to media and sponsors (20). In this context, adaptation cannot be reduced to cosmetic or technological interventions. Rather, it demands strategic reflection on the social role of sport in contemporary societies (21).

### Innovation and heritage sports: cultural negotiation and legitimacy

Building on these debates, the limits of dominant approaches to sport innovation become particularly visible in heritage sports. Much of the existing literature has focused on professionalized and globalized disciplines such as football, basketball, or tennis, privileging analytical frameworks oriented towards performance optimization, organisational efficiency, and commercial expansion (1, 3). Within these perspectives, technological innovations, regulatory adjustments, and marketing strategies are often conceptualized primarily as mechanisms aimed at maximizing performance, profitability, and market reach (2).

While these approaches have generated valuable insights, they are insufficient for understanding innovation processes in heritage sports such as Basque hand-ball, whose value transcends competitive outcomes and is deeply anchored in cultural frameworks, community relations, and territorial bonds. In such disciplines, innovation cannot be conceived solely as the introduction of technical or managerial improvements. Instead, it must be understood as a culturally negotiated process in which identity narratives, collective memories, and deeply rooted symbols play a constitutive role in shaping both acceptance and resistance to change (4).

From this standpoint, resistance to change should not be interpreted as irrational rejection or cultural conservatism, but as an expression of concern for the preservation of intangible heritage and for the continuity of meaning that sustains the sport across generations (22). Consequently, innovation in heritage sports requires culturally sensitive approaches that actively involve relevant stakeholders in the process of deliberation and design. Such participation is crucial for generating trust, reinforcing legitimacy, and fostering a shared perception of continuity between past practices and future adaptations.

Only through these negotiated and participatory processes is it possible to mitigate the perceived risk of identity loss and to design transformations that are viable not only from a technical or organisational perspective, but also from a symbolic and social one. This understanding provides the conceptual foundation for examining innovation in Basque hand-ball not as a problem of efficiency or spectacle alone, but as a governance challenge situated at the intersection of cultural sustainability, institutional legitimacy, and collective meaning.

### Regulatory transformations and the redesign of game elements

Within racquet and ball sports, organisational and regulatory changes have played a key role in the processes of transformation, visibility, and growth across various disciplines. From a theoretical perspective, these innovations have extended beyond technical rules to encompass the very structure of competitions, the configuration of the sporting spectacle, and the way in which such sports are made accessible to new audiences. The underlying rationale of these reforms often responds to a dual pressure: to adapt to contemporary consumption formats while preserving the internal coherence of the game.

Professional tennis offers a paradigmatic example. Since the early 21st century, the sport has undergone substantial modifications, such as the introduction of the final-set tie-break, the implementation of the electronic challenge system (Hawk-Eye), and the reduction of the competitive calendar—all aimed at enhancing the players’ physical sustainability and increasing the spectacle's dynamism (18). These transformations have reinforced perceptions of fairness and boosted entertainment value, thereby enhancing commercial and broadcast appeal.

In particular, scoring systems have generated substantial scholarly debate. Brams et al. (23) argue that proposals such as the Catch-Up Rule increase fairness and competitive tension. Complementarily, Szigeti (10) examined the statistical impact of experimental rules—such as removing the second serve after the first three points—and found that these adjustments increase unpredictability without compromising outcome fairness. In the case of Basque hand-ball, proposals such as adopting a unitary system up to 22 points or introducing specific penalties find empirical support in these approaches, sharing the underlying logic of promoting a more balanced, dynamic, and broadcast-friendly game.

Both collegiate and professional tennis have served as testing grounds for alternative formats, such as no-ad scoring and the temporary suppression of the second serve. These changes have proven effective in reducing match duration and increasing competitive uncertainty—two key factors in enhancing spectator experience (10, 24). In this vein, proposals in hand-ball aimed at adjusting the serve format to quicken the pace align with a broader international trend towards reconfiguring the temporal structures of sport.

Other sports have followed similar trajectories. In badminton, the 2006 reform of the scoring system—from 15 to 21 points under a rally point system—shortened match duration and enhanced dynamism, improving broadcast potential and reinforcing its presence at major international events such as the Olympic Games (17). Table tennis also underwent notable changes, including reducing sets to 11 points and introducing the white ball to improve television visibility. These reforms coincided with an increase in federative licenses across several European countries (25).

Padel, for its part, illustrates how an emerging sport can consolidate itself through organisational design geared towards accessibility. The implementation of short-format tournaments, clear rules, low infrastructure costs, and effective digital promotion strategies have enabled the sport's rapid expansion in both Europe and Latin America (26).

Taken together, these cases demonstrate that regulatory and structural transformations can act as levers for growth when designed with a deep understanding of the sport's internal logic and the evolving cultural and media demands. From a cultural-sociological perspective, the internal logic of a sport can be understood as the shared system of meanings and normative expectations through which sporting practices are recognised as legitimate within a given social context. Changes such as adopting shorter sets or reorganizing scoring systems have proven decisive in facilitating television broadcasting, attracting new audiences, and supporting institutional sustainability (25). Under these conditions, when such reforms respect the balance between tradition and novelty, they can legitimize change for traditional audiences, foster internationalization processes, and generate virtuous cycles of revitalization.

Thus, comparative experience indicates that adjustments to game elements are not merely technical modifications, but strategic interventions which, when well-directed, enable traditional sports to project themselves into the future without relinquishing their foundational identity.

### The delphi method in sports research and scenario planning in sport

The Delphi method is an iterative qualitative technique designed to reach expert consensus through a series of successive rounds involving anonymous consultation, controlled feedback, and structured synthesis of judgments. Its application is particularly appropriate in contexts where quantitative evidence is scarce or insufficient, and where expert knowledge—both explicit and tacit—becomes central to decision-making processes (11, 27). This approach holds particular relevance in traditional sports such as Basque hand-ball, where the lack of systematized data contrasts with a rich symbolic and practical heritage known in depth only to those embedded within the ecosystem (28).

In recent years, the use of the Delphi method in sport has expanded significantly, especially in defining public policy priorities, identifying professional competencies, and designing educational interventions. Costa (29), for instance, employed the method to determine key areas in the training of sports managers, while Quartiroli et al. (28) used it within sport psychology to reach consensus on best evaluative practices. Its versatility has also been demonstrated in studies analyzing stadium atmospheres (30), return-to-play criteria following injury (31), and the psychosocial benefits of school-aged sport participation (32).

Complementarily, scenario planning has emerged as a critical tool for strategic foresight in environments characterized by complexity, uncertainty, and rapid change. Unlike linear forecasting models that extrapolate from past trends, this approach enables the construction of divergent, plausible, and culturally situated narratives about alternative futures (12, 33). Its strength lies in integrating structural factors—such as institutional, economic, or demographic dynamics—with symbolic and affective dimensions that shape the social imaginary of sport.

Although its application in sport has been more limited than in fields such as economics or urban planning, recent research has highlighted its value. Merkel et al. (34) used scenario planning to anticipate structural changes in European professional football, while Scott and McBoyle (35) applied it to forecast the impact of climate change on snow and mountain sports. Its primary contribution lies in the capacity to generate shared future visions, integrating multiple dimensions—technical, cultural, and social—that influence the feasibility of decision-making.

The combination of Delphi and scenario analysis represents a particularly robust methodological strategy for studying minority or heritage sports, where regulatory uncertainty and cultural sensitivity complicate the application of conventional methods. Nowack et al. (13) argue that this integration allows expert consensus to be paired with strategic foresight, generating scenarios validated by collective judgement and responsive to contextual realities. In this sense, the Delphi method offers a reliable structure for gathering and contrasting expert knowledge anonymously, while scenario planning provides a framework for translating this knowledge into culturally legitimate courses of action (12, 33).

Within the field of sport science, Dašić (36) underscores the importance of adapting this combined methodology to enhance its applicability—through optimizing panel size, improving inter-round feedback quality, and managing dropout rates, particularly in studies involving heterogeneous stakeholders. Such refinements help strengthen the reliability of the process and increase its utility in complex contexts such as the organisational and symbolic transformation of traditional disciplines.

Drawing on this framework, the present study is guided by the following research questions:

RQ1. To what extent can prospective qualitative methods—specifically the combined use of the Delphi method and scenario planning—contribute to a deeper understanding and socially legitimate adaptation of traditional sports in contemporary contexts?
RQ2. Which elements of the professional Basque hand-ball game are perceived by sport ecosystem experts as both spectacular and viable, and how can these elements inform future regulatory or organisational transformations without compromising the sport's cultural identity?

## Methods

This study was methodologically structured through the combination of the qualitative Delphi method and scenario planning techniques. This integration enabled the systematic collection of expert knowledge—both explicit and tacit—from key actors within the Basque hand-ball ecosystem, as well as the projection of plausible futures through a culturally sensitive and technically feasible lens (11–13, 28). The entire process was developed over three successive waves of structured consultation, following the classical Delphi approach characterized by controlled feedback and participant anonymity.

The research began with a structural analysis to identify key variables for constructing initial scenarios. In parallel, a qualitative analysis of the game was conducted from the perspective of stakeholders involved in different modalities of ball, including “remonte”, “pala”, “cesta punta”, and hand-ball. Interviews carried out during this exploratory phase provided a comparative perspective across disciplines and helped identify innovation mechanisms previously implemented that could be adapted to the specific context of professional hand-ball.

From this groundwork, six core elements of the game were identified as susceptible to transformation: pre-match warm-up, selection of equipment before and during the match, service format, scoring system, and game temporality. These elements were used as a basis for scenario construction, applying a foresight methodology structured around two analytical axes: game spectacularity and competitive equity.

The combination of these axes gave rise to four differentiated scenarios. One scenario proposed matches played to 22 consecutive points, prioritizing spectacularity but compromising competitive balance. Another suggested a system of 11-point games, favoring competitive fairness while potentially sacrificing dramatic tension. A third, more ambitious scenario proposed structuring matches into 6-point games to maximize both spectacularity and equity. Lastly, a fourth scenario focused on 10-point games, aiming to balance both dimensions without fundamentally altering the traditional logic of the sport.

These scenarios were then assessed by an expanded panel of experts in a second Delphi round, which focused on evaluating their technical feasibility, symbolic coherence with traditional practices, and potential acceptance among the various stakeholders in the ecosystem. A third round of evaluation further refined the highest-rated proposals, prioritizing those with the strongest consensus and greatest implementation potential.

This methodological process not only enabled the generation of culturally legitimate proposals for change but also facilitated their translation into concrete measures. In fact, several of the recommendations emerging from this process have subsequently been introduced within the Torneo Bizkaia, demonstrating the practical applicability of the approach in a professional competition context. The incorporation of these measures highlights how strategic reflection, situated knowledge, and expert deliberation can inform adjustments to contemporary competitive structures while maintaining coherence with the sport's traditional foundations.

### Procedure and participant selection for successive delphi waves

The Delphi methodology requires collecting expert opinions and assessments regarding potential future scenarios. The selection of the expert panel was carried out using purposive sampling, complemented by snowball sampling techniques, in order to ensure representation across a range of professional profiles linked to Basque ball.

In the first round, the objective was to contextualize and understand changes that had occurred in other modalities of Basque ball (see Table 1).

**Table 1:** Participant sample distribution—first delphi wave.

Professional background	1st wave
Professional hand-ball coordinator	1
Technical coordinator for professional remonte	1
Technical coordinator for professional pala	1
Professional hand-ball players	2
Total	5

In a second wave (see Table 2), a broader group of stakeholders involved in the world of hand- ball was recruited, with the aim of identifying key elements that could serve as the foundation for developing future scenarios. A questionnaire was designed as a supporting tool for the interviews conducted during the first wave, in which the different scenarios were presented. This wave included the participation of 23 experts (22 men and 1 woman). The underrepresentation of women in the sample should be interpreted not as a sampling bias but as a manifestation of the structural inequality embedded in Basque hand-ball, where the lack of a professional women's league constrains women's access to elite competitive pathways and, consequently, their visibility within expert and professional domains.

**Table 2:** Distribution of participant sample in the 2nd and 3rd delphi waves.

Professional background	2nd wave	3rd wave
Sports directors	3	4
Organisers of elite amateur hand-ball tournaments	3	
Coaches	2	
Sport psychologist	1	
Active professional players	4	4
Players’ association representative	1	
Referee	1	1
Academics	1	2
Trainers	2	2
Media experts	1	1
Former players	2	2
Others	2	3
Total	23	19

For the third wave, a second questionnaire was developed to gather expert evaluations on the feasibility of the proposed scenarios. Table 2 shows the distribution of participants in the third Delphi wave according to their professional background.

### Procedure

The process began with a first wave focused on conducting semi-structured interviews with experts from the hand-ball ecosystem. The objective was to explore perceptions, previous experiences, and proposals for adjustment regarding the current competitive model. These interviews were guided by a protocol organized around three main axes: the evaluation of the existing system; lessons learned from changes in other traditional pelota modalities; and potential modifications to specific elements of the game. The interviews, lasting between 60 and 90 min, were audio-recorded with informed consent and analyzed through interpretative qualitative analysis. Coding revealed two major vectors of transformation: the pursuit of greater spectacle and the desire to enhance competitive fairness between players. The intersection of these two axes structured the configuration of four scenario frameworks, each centered on different components of the game, such as the scoring system, game duration, or serve regulations.

Based on these findings, the second wave was designed around a structured questionnaire that operationalized key elements of the scenarios into 24 items, each representing a specific measure. These items were grouped into thematic blocks (e.g., warm-up, equipment selection, serve rules, or match duration) and rated using a 5-point Likert scale, accompanied by open fields for qualitative justifications.

The final version of the questionnaire, validated through a pilot test with two experts, was mostly administered in face-to-face sessions, during which participants completed the instrument alongside a member of the research team, who facilitated the process and occasionally encouraged brief interpretive discussions. A total of 23 valid responses were collected.

Data were analysed using descriptive measures of central tendency. A mean threshold of ≥3.5 was applied as an operational criterion for interpretation rather than as a statistical test of consensus. On a five-point Likert scale, the value of 3 represents a neutral or ambivalent position, while values of 4 and 5 indicate a positive evaluation. Establishing the cut-off at ≥3.5 therefore made it possible to differentiate items showing a clear tendency towards positive endorsement from those reflecting neutrality or disagreement. This threshold was selected to balance interpretative clarity with sensitivity to the exploratory nature of the study and the limited size of the expert panel. The criterion was applied consistently across items to facilitate the comparative construction of innovation scenarios, without implying the existence of definitive or normative consensus.

Measures with a mean rating of 3.5 or higher were considered positively consensual, those below 2.5 were deemed rejected, and items between these thresholds were interpreted as ambiguous. This analysis was complemented by exploring differences between professional profiles, particularly between active athletes and organisational actors. Based on this structured evaluation, the scenarios were refined: proposals with low support were removed, others were adjusted based on qualitative feedback, such as preserving symbolic elements of the game, and highly consensual measures were maintained. The result was a refined version of the four scenarios, better aligned with the field's expectations, needs, and cultural sensitivities.

The third wave consisted of a final validation exercise of the revised scenarios, through an online questionnaire sent to the 23 experts who had participated in the previous round. This new instrument briefly presented the four revised scenarios and asked respondents to assess their feasibility for short-to-medium-term implementation, using the same 1-to-5 scale. An open-ended section allowed participants to identify obstacles, necessary conditions, or recommendations to facilitate implementation of the most viable scenario according to their judgement. The questionnaire was accompanied by a summary report with aggregated results from the second wave, thus adhering to the controlled feedback principle of the Delphi method. This strategy enabled informed decision-making while maintaining respondent anonymity and preventing direct peer influence.

Nineteen experts responded within the established timeframe. Their responses were analyzed both in aggregate and disaggregated by professional profile, allowing for observation of how consensus was distributed across different stakeholder groups. Quantitative scores were complemented by thematic coding of the most repeated arguments, which provided interpretive insight into the reasons behind support or rejection of each scenario. Overall, this third phase enabled not only the identification of the most viable scenario, but also the consolidation of a robust and informed consensus regarding the technical and cultural conditions necessary for a potential transformation of the professional hand-ball competitive model.

Finally, once all data were collected, a triangulation process was carried out between qualitative and quantitative findings, complemented by the identification of emergent aspects not captured in the questionnaire items. Throughout the process, field notes were taken and analysis matrices were developed to register emergent ideas. For instance, in the second wave, some experts spontaneously offered useful comparisons such as “this resembles the tennis tie-break” or “it could attract younger audiences, like what happened with “cesta punta” in Miami.” These contributions were systematically documented to enrich the interpretation of results.

All participation was voluntary, anonymous, and could be withdrawn at any point. Confidentiality of responses was guaranteed at all times —particularly important given the institutional profile of some participants. The study was conducted in accordance with the ethical principles outlined in the Declaration of Helsinki (World Medical Association, 2013) and was approved by the Ethics Committee of the University of Deusto (ref. ETK-31/23-24).

## Discusssion

The results of this study are organized around two analytical axes. First, they examine proposed changes according to specific elements of the game—such as the scoring system, serve regulations, and equipment selection—evaluating their perceived contribution to spectacularity and their technical feasibility. Second, they analyse how these evaluations vary across professional profiles within the Basque hand-ball ecosystem, highlighting differentiated patterns of acceptance among players, coaches, referees, media professionals, and organisational actors.

### Proposed changes by game element

Regarding the scoring system, the highest-rated format was matches played as the best of three games to 10 points, with a five-point tiebreaker. This configuration allows for a moderate increase in total points (47 vs. the current 43) without substantially altering the traditional structure of the game. Consistent with previous research on heritage sports, such reforms appear to be more readily accepted when they preserve the internal logic of play while enhancing narrative clarity and competitive balance (4, 18). By contrast, more disruptive proposals—such as requiring a two-point difference to win—were rejected due to their incompatibility with broadcast time constraints (20).

Among the highest-rated measures were: allowing the starting player to choose the ball (spectacle: 4.00; feasibility: 4.52), imposing a 25-second limit between serves (4.14; 3.95), and introducing progressive penalties for infractions (4.33; 4.05). Regarding ball selection, following O'Boyle and Shilbury (37), it is recommended that this be the responsibility of a neutral body to avoid conflicts of interest, within an ethical governance framework (38). During the match, the optimal scenario envisaged a maximum of 15 s for ball changes, ensuring the pace of play without compromising player autonomy.

Taken together, the highest-rated proposals point to a pattern of legitimacy-preserving innovation rather than a pursuit of spectacle at any cost. Experts favoured low-disruption procedural adjustments—such as tighter serve timing, clearer sanction ladders, and regulated ball-change windows—that enhance coordination and readability while leaving intact the sport's internal logic and symbolic grammar. In heritage sports, such reforms are more likely to be accepted when they are perceived as internal refinements rather than identity-threatening ruptures (39–41).

As for the serve, a proposal was made to advance it to the 4½ line to lengthen rallies and balance competitive opportunities, following the logic of targeted interventions that improve the spectacle without compromising the sport's identity (23). Likewise, the use of initial draws and alternating serves by game reinforces meritocratic principles linked to accumulated performance, aligning with strategic planning approaches in sport (12). These measures correspond to conditions identified as critical for innovation acceptance, namely compatibility with tradition and perceived legitimacy (2, 42).

Proposals receiving intermediate ratings (means between 3 and 4) included: allowing warm-ups on the main court when available (spectacle: 3.62–3.67; feasibility: 3.24–3.38), ball selection without prior testing (3.76; 3.95), serve adjustments for doubles matches (3.48; 3.95), a unitary scoring system to 22 points (3.67; 4.24), and the introduction of strategic breaks at points 12 and 18 (3.52; 3.29). On the topic of warm-ups, experts agreed on the need for formalization, suggesting scenarios with a standard 10-minute period or adjustments based on ambient temperature. These views align with the recommendations of Amer et al. (33) and recent literature on athletic performance in variable environments (28).

Further proposals included restrictions on voluntary timeouts and the interval between serves, incorporating a progressive penalty system inspired by other sports such as tennis or badminton (4, 20). While positively evaluated, these measures require broad organisational consensus and careful implementation. As Landeta (43) emphasizes, in disciplines with strong symbolic foundations, stakeholder participation is crucial to avoid fears of losing authenticity. Such proposals occupy an intermediate zone that is particularly fertile for the co-design of shared scenarios, as argued by authors such as Schoemaker (12) and Nowack et al. (13).

At the opposite end of the spectrum, proposals that directly altered highly ritualized or symbolic elements—such as imposing a single ball per game, prohibiting ball repetition, increasing serve distance, or eliminating player-requested timeouts—were consistently rejected. These measures were perceived as disrupting foundational structures of the game, confirming previous findings that changes affecting ritualized practices tend to generate resistance in heritage sports (4, 18, 20).

Overall, the results indicate that the most viable reforms are not those that maximize disruption, but those that successfully harmonize dynamism, competitive fairness, and respect for tradition. This reinforces the value of combining the Delphi method with scenario planning to capture the symbolic, organisational, and cultural complexity of innovation processes in heritage sports (28, 33).

### Scenario evaluations according to the actors involved in basque hand-ball

Disaggregated analyses revealed clear differences in how reform scenarios were evaluated across professional profiles, reflecting distinct positional rationalities within the sporting ecosystem (44). Commercial and media actors tended to favor stable and standardized formats—most notably the current 22-point system—due to its compatibility with production and broadcasting requirements. By contrast, players, coaches, and strength and conditioning staff consistently preferred the 10- and 11-point formats, which were perceived as more manageable in terms of workload, rhythm, and tactical coherence (45, 46).

Referees rated the 22-, 11-, and 10-point formats similarly (mean = 4), while clearly rejecting the 6-point format (mean = 2). This cautious stance towards reforms that increase game variability is consistent with evidence from sports such as tennis and cricket, where rule changes often generate scepticism due to their implications for rule interpretation and officiating stability (10).

From an academic perspective, the 11-point format emerged as the most viable option (mean = 4), whereas both the 22- and 6-point formats were considered least viable (mean = 2). This assessment reflects a systemic evaluation of reform, attentive to the broader structural effects that regulatory changes may have on gameplay coherence and the overall sporting experience (47).

Strength and conditioning coaches showed a clear preference for the 10-point format (mean = 4.5), likely due to the structured pauses it introduces, which facilitate load management without disrupting game rhythm. This interpretation aligns with research highlighting the importance of effort–recovery micro cycles in high-intensity sports such as hand-ball (46).

Media professionals identified the current 22-point system as the most viable format (mean = 5), reflecting a preference for standardized structures that facilitate television production and broadcasting. Similar dynamics have been observed in tennis, where broadcaster demands have driven the introduction of short formats such as *Fast4* to sustain audience engagement (48).

Active professional players favoured the 10- and 11-point formats (mean = 3.25) and assigned the lowest evaluation to the existing 22-point system (mean = 1.75), reflecting a logic of performance optimization and strategic manageability. Elite amateur players displayed a comparable pattern, strongly supporting the 10-point format (mean = 4) and rejecting the 6-point option (mean = 1.5). Former players rated the 10-point scenario even more favourably (mean = 4.5), a tendency associated with accumulated strategic insight over extended sporting careers (45).

Taken together, these differentiated evaluations make it possible to identify regulatory formats that are simultaneously perceived as viable and consistent with the internal logic of Basque hand-ball across stakeholder groups. The results reveal a clear convergence between coaches and players—both active and retired—around the 10-point format as the most viable option, while the 6-point format is consistently regarded as the least viable across profiles. This pattern mirrors findings from participatory reform processes in sports such as netball and volleyball, where the legitimacy of regulatory change depends on inclusive dialogue that integrates technical, symbolic, and organisational considerations across stakeholder groups (49).

Profile-based differences are analytically meaningful because they reflect distinct legitimacy audiences within the sporting ecosystem. Commercial and media actors tend to prioritise stability, format standardisation, and predictable production constraints, whereas players and performance-oriented staff emphasize competitive manageability, workload rhythm, and tactical coherence. Rather than constituting mere preference variation, these divergences point to a governance challenge of representation and justification, whereby reform proposals must be rendered acceptable to constituencies holding different authority claims over what constitutes a legitimate change (50). In this sense, innovation in heritage sport depends less on average support than on the capacity of reforms to be justified through recognised governance principles—such as transparency, role-appropriate influence, and safeguards against capture—while sustaining democratic representational credibility (51). This positions stakeholder divergence itself as a substantive analytical finding, highlighting the arenas in which deliberation and institutional design are required for reforms to achieve procedural legitimacy, particularly in heritage sport contexts where legitimacy is both cultural and operational (52).

A counterfactual perspective highlights the importance of participatory approaches in heritage sports, as top-down innovation risks prioritising efficiency or commercial visibility over cultural legitimacy, potentially generating resistance and eroding trust. In contexts where sporting practices carry strong symbolic meanings, deliberative methodologies such as the Delphi method operate not only as data-collection tools but also as governance instruments that mediate between tradition and change, fostering innovation pathways perceived as legitimate and culturally sustainable.

More broadly, the observed evaluation patterns reflect actors’ positions within the sporting field and the coexistence of multiple rationalities. The most viable scenarios align with a logic of sustainable innovation (2), combining heritage preservation with contemporary demands for spectacle, fairness, and good governance. Beyond its empirical context, this study contributes an analytically grounded framework for examining innovation in heritage sports, conceptualising it as a culturally negotiated process shaped by legitimacy, governance arrangements, and stakeholder participation. By integrating the Delphi method with scenario analysis, the research offers a transferable approach for understanding not only which innovations are favoured, but why certain pathways of change are perceived as acceptable within culturally embedded sporting contexts.

## Conclusions

In a sporting landscape increasingly pressured by the need to renew formats without eroding the heritage value of traditional disciplines, this study has demonstrated that combining the Delphi method with scenario planning constitutes an effective, deliberative, and culturally sensitive methodological tool. Applied to the case of Basque hand-ball, this approach enabled the construction of reform proposals that are not only technically feasible and operational but also enjoy symbolic legitimacy and acceptance among the diverse actors within the sporting ecosystem.

Unlike other consultative processes designed to validate decisions already made, this research facilitated the collective production of situated knowledge. From the initial interviews to the final consensus-building phase, the procedure promoted informed dialogue among diverse profiles—players, coaches, referees, media professionals, and organisers—integrating perspectives that typically operate in fragmented ways. In line with Schoemaker (12), Nowack et al. (13), and Quartiroli (28), the study reaffirms the transformative potential of prospective qualitative methods when they succeed in articulating technical expertise with sociocultural context.

The results show that regulatory innovation in heritage sports can be designed from within, respecting the symbolic elements that shape the meaning of the game. Reforms such as shortening match formats, implementing progressive penalties for delays between serves, or neutralizing the selection of game materials were especially well valued for their ability to enhance spectacle without altering the internal logic of the sport. The subsequent adoption of several of these measures in the current configuration of the professional hand-ball league provides empirical support for the relevance and applicability of the approach.

Moreover, the segmented analysis by professional profile reveals that preferences regarding reform are mediated by the position each group occupies within the sporting field. While players and coaches prioritise aspects related to physical and tactical efficiency, media professionals and organisers tend to favor narrative continuity and organisational stability. Rather than constituting an obstacle, this diversity underscores the need for participatory governance frameworks capable of integrating multiple rationalities and managing conflict through mutual recognition (44, 49).

This work confirms that prospective qualitative methods not only enable us to understand future imaginaries in traditional sports disciplines but also serve as operational tools for intervening in them in a realistic, legitimate, and transformative manner. Innovation, in this context, is not a rupture or a top-down imposition, but a negotiated construction of possible futures that balances tradition, competitive fairness, and media adaptability.

Beyond its local impact, the proposed methodology is transferable to other racket and ball sports facing similar challenges of modernization and may be especially valuable for sports federations, public administrations, and local entities seeking to design context-sensitive, technically sound, and culturally viable policies.

That said, the study presents several important limitations. The selection of the panel, although deliberately diverse, does not guarantee full representativeness of the hand ball ecosystem. The qualitative and prospective nature of the study precludes generalizations without additional empirical validation. Finally, the scope has been limited to the Basque context, and its applicability to other regions or sports should be assessed with caution and with due attention to their specific cultural and organisational characteristics.

## Data Availability

The raw data supporting the conclusions of this article will be made available by the authors, without undue reservation.
