# Supplementary material for: Innovation in traditional sport: applying the delphi method to strategic designs in basque hand-ball
Source: Front Sports Act Living. 2026 Jan 30;8:1704042. doi: 10.3389/fspor.2026.1704042 (PMC12903272; doi:10.3389/fspor.2026.1704042)
Supplement: Supplementary file 1 [file Table1.docx]

**Appendix A. Survey instrument**

This appendix presents the full questionnaire used in the study. The instrument was designed to collect expert assessments on potential regulatory and organisational changes aimed at increasing the attractiveness and spectacularity of Basque handball. All evaluative items were measured on a five-point Likert scale (1 = very low / strongly disagree; 5 = very high / strongly agree). For analytical purposes, items were grouped into thematic blocks corresponding to key dimensions of the game (warm-up procedures, material selection, serve, scoring system, and temporal regulation).

**A.1. Sociodemographic and background information**

- **Q27.** Indicate the field in which you are involved in Basque handball.
- **Q28.** Indicate your sexual identity.
- **Q26.** Indicate the number of years you have been involved in Basque handball or describe your career trajectory.
- **Q29.** Indicate your age.

**A.2. General relevance of game elements for spectacularity**

- **Q5_1.** To what extent do you consider warm-up procedures relevant for increasing the spectacularity of the game?
- **Q5_2.** To what extent do you consider material selection relevant for increasing the spectacularity of the game?
- **Q5_3.** To what extent do you consider the serve relevant for increasing the spectacularity of the game?
- **Q5_4.** To what extent do you consider the scoring system (tanteo) relevant for increasing the spectacularity of the game?
- **Q5_5.** To what extent do you consider time limitation of the match relevant for increasing the spectacularity of the game?

**A.3. Warm-up – Feasibility**

- **Q4_1.** Allocating 5 minutes on the main court for warm-up when a secondary court is available.
- **Q4_2.** Allocating 10 minutes on the main court for warm-up when no secondary court is available.
- **Q4_3.** Conducting warm-up activities before and during the match until both parties agree.

**A.4. Warm-up – Contribution to spectacularity**

- **Q8_1.** Allocating 5 minutes on the main court for warm-up when a secondary court is available.
- **Q8_2.** Allocating 10 minutes on the main court for warm-up when no secondary court is available.
- **Q8_3.** Conducting warm-up activities before and during the match until both parties agree.

**A.5. Material selection – Contribution to spectacularity**

**Pre-match**

- **Q10_1.** Use of a dynamic ball reaching more than three court sections.
- **Q10_2.** Pre-match press conference.
- **Q10_3.** Live broadcast of the match.
- **Q10_4.** Ball selection by the selector without considering players or court characteristics.

**During the match**

- **Q10_5.** Selecting the ball without testing it.
- **Q10_6.** Inability to choose the ball whenever a “tanto” is scored.
- **Q10_7.** Playing one game (“joko”) with the same ball.
- **Q10_8.** Inability to reuse the same ball in different games.
- **Q10_9.** The serving player chooses the ball.
- **Q10_10.** Allowing ball changes at any time.
- **Q10_11.** No time limit for testing the ball.
- **Q10_12.** Option to test the ball against the front wall.
- **Q10_13.** Choosing the ball during the match without testing it.

**A.6. Material selection – Feasibility**

**Pre-match**

- **Q12_1.** Use of a dynamic ball reaching more than three court sections.
- **Q12_2.** Pre-match press conference.
- **Q12_3.** Live broadcast of the match.
- **Q12_4.** Ball selection by the selector without considering players or court characteristics.

**During the match**

- **Q12_5.** Selecting the ball without testing it.
- **Q12_6.** Inability to choose the ball whenever a “tanto” is scored.
- **Q12_7.** Playing one game (“joko”) with the same ball.
- **Q12_8.** Inability to reuse the same ball in different games.
- **Q12_9.** The serving player chooses the ball.
- **Q12_10.** Allowing ball changes at any time.
- **Q12_11.** No time limit for testing the ball.
- **Q12_12.** Option to test the ball against the front wall.
- **Q12_13.** Choosing the ball during the match without testing it.

**A.7. Serve – Contribution to spectacularity**

- **Q13_1.** Same player serves throughout the game.
- **Q13_2.** In doubles, serve decided by the pair.
- **Q13_3.** Time limit between serves (25 seconds).
- **Q13_4.** Increased serving distance.
- **Q13_5.** Penalty for exceeding the serving time limit.
- **Q13_6.** Forward player allowed to intervene in the serve.
- **Q13_7.** Single serve only.
- **Q13_8.** Maintaining current serving distances.
- **Q13_9.** No time limit for serving.
- **Q13_10.** In games (“jokos”), the loser of the previous game serves first.
- **Q13_11.** Alternating serve in games.
- **Q13_12.** Same player serves throughout each game.
- **Q13_13.** Serve bounce closer to the front wall and passing closer to the rebound.
- **Q13_14.** Tie-break with mandatory serving by both forwards and defenders.
- **Q13_15.** Time limit of 25 seconds with no possibility for the opposing player or pair to test the ball.

**A.8. Serve – Feasibility**

- **Q14_1.** Same player serves throughout the game.
- **Q14_2.** In doubles, serve decided by the pair.
- **Q14_3.** Time limit between serves (25 seconds).
- **Q14_4.** Increased serving distance.
- **Q14_5.** Penalty for exceeding the serving time limit.
- **Q14_6.** Forward player allowed to intervene in the serve.
- **Q14_7.** Single serve only.
- **Q14_8.** Maintaining current serving distances.
- **Q14_9.** No time limit for serving.
- **Q14_10.** In games (“jokos”), the loser of the previous game serves first.
- **Q14_11.** Alternating serve in games.
- **Q14_12.** Same player serves throughout each game.
- **Q14_13.** Serve bounce closer to the front wall and passing closer to the rebound.
- **Q14_14.** Tie-break with mandatory serving by both forwards and defenders.
- **Q14_15.** Time limit of 25 seconds with no possibility for the opposing player or pair to touch the ball.

**A.9. Scoring system (tanteo) – Contribution to spectacularity**

- **Q17_1.** Single system (match to 22 points).
- **Q17_2.** Best of five games in matches to 6 points, with a final game to 5.
- **Q17_3.** Best of three games in matches to 11 points, with a final game to 5.
- **Q17_4.** Best of five games with a tie-break in the final game.

**A.10. Scoring system (tanteo) – Feasibility**

- **Q20_1.** Single system (match to 22 points).
- **Q20_2.** Best of five games in matches to 6 points, with a final game to 5.
- **Q20_3.** Best of three games in matches to 11 points, with a final game to 5.
- **Q20_4.** Best of five games with a tie-break in the final game.

**A.11. Time limitation of the match – Contribution to spectacularity**

- **Q21_1.** Fixed total match duration (1 hour and 15 minutes).
- **Q21_2.** Fixed breaks at points 12 and 18.
- **Q21_3.** Elimination of player-requested breaks.
- **Q21_4.** Four 2-minute breaks at the end of each game.
- **Q21_5.** Two 2-minute breaks at the end of each game.
- **Q21_6.** Player-requested breaks allowed during the game.
- **Q21_7.** Playing without any time limit.
- **Q21_8.** Maximum real playing time per game (15 minutes).
- **Q21_9.** Final game with a maximum of 10 minutes of real playing time.
- **Q21_10.** Penalty for exceeding time limits.

**A.12. Time limitation of the match – Feasibility**

- **Q24_1.** Fixed total match duration (1 hour and 15 minutes).
- **Q24_2.** Fixed breaks at points 12 and 18.
- **Q24_3.** Elimination of player-requested breaks.
- **Q24_4.** Four 2-minute breaks at the end of each game.
- **Q24_5.** Two 2-minute breaks at the end of each game.
- **Q24_6.** Player-requested breaks allowed during the game.
- **Q24_7.** Playing without any time limit.
- **Q24_8.** Maximum real playing time per game (15 minutes).
- **Q24_9.** Final game with a maximum of 10 minutes of real playing time.
- **Q24_10.** Penalty for exceeding time limits.

Principio del formulario

Final del formulario

Final del formulario
